# Reduction of quaternary ammonium-induced ocular surface toxicity by emulsions: an in vivo study in rabbits

**Published:** 2008-01-31

**Authors:** H. Liang, F. Brignole-Baudouin, L. Rabinovich-Guilatt, Z. Mao, L. Riancho, M.O. Faure, J.M. Warnet, G. Lambert, C. Baudouin

**Affiliations:** 1 Department of Toxicology, Faculty of Biological and Pharmacological Sciences and; 2 INSERM, UMR S 872, Cordeliers, University Paris Descartes, France; 3 Department of Ophthalmology III, Quinze-Vingts National Ophthalmology Hospital, Paris, and Ambroise Paré Hospital, APHP, University of Versailles, France; 4 Novagali Pharma SA, 1 rue Pierre Fontaine, 91058 Evry, France

## Abstract

**Purpose:**

To evaluate and compare the toxicological profiles of two quaternary ammonium compounds (QAC), benzalkonium chloride (BAK), and cetalkonium chloride (CKC), in standard solution or cationic emulsion formulations in rabbit eyes using newly developed in vivo and ex vivo experimental approaches.

**Methods:**

Seventy eyes of 35 adult male New Zealand albino rabbits were used in this study. They were randomly divided into five groups: 50 µl of phosphate-buffered saline (PBS), PBS containing 0.02% BAK or 0.002% CKC (BAK Sol and CKC Sol, respectively), and emulsion containing 0.02% BAK or 0.002% CKC (BAK Em and CKC Em, respectively) were applied to rabbit eyes 15 times at 5-min intervals. The ocular surface changes induced by these eye drops were investigated using slit-lamp examination, flow cytometry (FCM), impression cytology (IC) on conjunctiva, and corneal in vivo confocal microscopy (IVCM). Standard immunohistology in cryosections was also examined for cluster of differentiation (CD) 45+ infiltrating and terminal deoxynucleotidyl transferase-mediated dUTP-nick end labeling (TUNEL)+ apoptotic cells.

**Results:**

Clinical observations and IVCM showed that the highest toxicity was induced by BAK Sol, characterized by damaged corneal epithelium and a high level of inflammatory infiltration. BAK Em and CKC Sol presented moderate effects, and CKC Em showed the lowest toxicity with results similar to those of PBS. Conjunctival imprints analyzed by FCM showed a higher expression of RLA-DR and TNFR1 markers in BAK Sol-instilled eyes than in all other groups, especially at 4 h. Immunohistology was correlated with in vivo and ex vivo findings and confirmed this toxicity profile. A high level of infiltration of CD45+ inflammatory cells and TUNEL+ apoptotic cells was observed in limbus and conjunctiva, especially in QAC solution-receiving eyes compared to QAC emulsion-instilled eyes.

**Conclusions:**

The acute administration of 15 instillations at 5 min intervals was a rapid and efficient model to assess quaternary ammonium toxicity profiles. This model showed the highest toxicity, induced by the BAK solution, and the lowest level of toxicity, induced by the CKC emulsion. These in vivo and ex vivo experimental approaches demonstrated that ocular surface toxicity was reduced by using an emulsion instead of a traditional solution and that a CKC emulsion was safe for future ocular administration.

## Introduction

During the past few years, several oil-in-water emulsions have been introduced on the market for the treatment of dry eye syndrome and are used as tear substitutes or vehicles for active compounds [1-4]. These oil-in-water emulsions can be visualized as tiny oil droplets suspended within an aqueous phase, and they are particularly suitable for dry eye syndromes because of the oil supplementation to the tear film. Oil-in-water emulsions can be charged positively (for example, the surface of the tiny droplets becomes cationic) by adding quaternary ammonium compounds (QACs) as cationic agents [1,2,5]. This cationic emulsion technology presents the advantage of the electrostatic attraction between the positively charged emulsion with the negatively charged ocular surface (cornea and conjunctiva), which allows longer residence time and hence, an improved ocular bioavailability of the active compound [1-5].

However, the use of QACs as cationic agents at relatively high concentrations in emulsion raises concerns regarding their toxicological profile. As the most commonly used preservative [6], benzalkonium chloride (BAK), has shown its high level of toxicity in vitro and ex vivo by stimulating epithelial cell death [7,8], acting as pro-inflammatory or pro-apoptotic mediators [9], inducing oxidative stress [10-12], and significantly altering the precorneal mucins [13]. In vivo, these iatrogenic effects were most particularly found with the eye drops used for treating long-term pathologies such as glaucoma. The analysis of conjunctival epithelium using flow cytometry (FCM) showed increased human leukocyte antigen (HLA) DR class II antigens, interleukin (IL) synthesis such as IL-6, IL-8, IL-10 [14], and the involvement of both T helper (Th)1 and TH2 systems through the overexpression of CC chemokine receptor (CCR) 5 and CCR4 in the conjunctiva of long term-treated glaucomatous patients [15,16]. Moreover, BAK-induced conjunctival fibrosis is considered a relevant risk factor for glaucoma surgery failure [17].

Indeed, several papers have reported that the efficacy of different preservatives including BAK is attenuated when incorporated within an oil-in-water emulsion [18-20]. In emulsions, high concentrations of antimicrobial agents were needed to achieve effective preservative activity [21]. In emulsion, BAK partitioned preferentially in the oil phase, resulting in only approximately 1.2% free BAK in solution in the aqueous phase [22]. The antimicrobial activity and the correlated toxicity are driven by the free preservative in solution whereas the emulsion-bound preservative seems to lose its efficacy.

BAK is composed of a mixture of alkylbenzyldimethyl ammonium chlorides bearing various alkyl chain lengths, each one with a different water solubility and water-octanol partition coefficient [23,24]. For the development of cationic emulsions in ophthalmology, the use of QAC for their cationic property rather than their preservative effect is being considered. We suggested the use of lipophilic cetalkonium chloride (CKC), one of the longest alkyl-chain BAK components, as a cationic agent in ophthalmic emulsions. With a highly lipophilic QAC, the distribution between the oil and aqueous phases of the emulsion is modified because of the affinity toward the oil phase, further favoring the cationic agent role over the preservative role.

It is important for further clinical developments to study the toxicological profiles of these new BAK or CKC-cationic emulsions and to compare them with solutions. Over the past few years, our group has developed new in vivo tools to explore the ocular surface of animal models. In vivo confocal microscopy (IVCM) offers a high definition of histological-like images that correspond very well with standard immunohistology of healthy and pathological conjunctivae or corneas [25-28]. It can be used repeatedly in vivo to follow a disease course or a healing process. Moreover, the evaluation of impression cytology (IC) specimens with FCM has been widely used to detect inflammation, apoptosis, or TH1/TH2 profiles in patients [14-16], in rabbit conjunctivitis, and in rat ocular toxicity models [25,27,28]. Class II HLA-DR antigens and tumor necrosis factor (TNF)-related markers were thus found to be involved in toxicological or inflammatory pathways of the ocular surface [14-16,25].

In this study, we combined two new investigative methods (IVCM for in vivo tissues images and IC for ex vivo epithelium inflammatory marker expression) in correlation with standard immunohistology for deep infiltration and apoptosis to assess the toxicological effects of BAK/CKC emulsion/solution formulations on the ocular surface of rabbits. We chose an experimental model described by Ichijima, consisting of 15 successive instillations in rabbit eyes at 5-min intervals [29]. This model presents the advantages of inducing a toxic injury in a relatively short time and of emphasizing the effects of standard concentrations of compounds in which its toxicity could only be assessed over the long-term in standard instillation conditions. Our objectives were to evaluate the interest of QAC-containing emulsions compared to QAC-containing solutions, to compare BAK and CKC toxicity, and to assess the ocular safety of the newly developed CKC cationic emulsion.

## Methods

### Animals and eye drop treatments

All experiments were conducted in accordance with the ARVO Statement for the Use of Animals in Ophthalmic and Vision Research. Male albino rabbits (New Zealand; two to three kilogram) were used. Before all experiments, the ocular surface integrity was examined by slip-lamp microscopy. A mixture of ketamine (35 mg/kg; Imalgène 500; Merial, Lyon, France) and xylazine (5 mg/kg; Bayer, Puteaux, France) was used to anesthetize the animals. Each group was composed of seven rabbits: five rabbits were used for clinical and IVCM observation, conjunctival imprints collection at hour (H) 4, day (D) 1, D4, and D7; two rabbits from each treatment were sacrificed for immunohistological procedures at D1, a time point chosen for the maximal inflammatory infiltration according to a preliminary seven-day study (data not shown).

**Table 1:** In vivo confocal microscopy scale for the evaluation of ocular toxicity in the cornea, the limbus, and the conjunctiva (maximum score: 40)

Tissue	Ocular surface damage		IVCM scale
	Property	Grade	
Superficial epithelium (10)	DESQUAMATION	Partial	**2**
		Total important	**4**
	SHAPE/SIZE	anisocytosis, microcytosis, macrocytosis, irregular shape, edematous cells, swollen cells, loss of cell borders	**2**
	REFLECTIVITY	abnormal reflectivity patterns: hyperreflective cells, nuclei visible in hyperreflective cells or not	**2**
	INFLAMMATION	presence of inflammatory infiltration	**2**
Basal epithelium (10)	DISORGANIZATION		**2**
	INFLAMMATORY INFILTRATION	0>slight >100 cells/mm^2^	**2**
		50>mild> 100 cells/mm^2^	**4**
		100>moderate> 200 cells/mm^2^	**6**
		severe> 200 cells/mm^2^	**8**
Anterior stroma (10)	DISORGANIZATION		**2**
	INFLAMMATORY INFILTRATION	0>slight >50 cells/mm^2^	**2**
		50>mild > 100 cells/mm^2^	**4**
		100>moderate> 200 cells/mm^2^	**6**
		severe> 200 cells/mm^2^	**8**
Limbus and conjunctiva (10)	PRESENCE OF CAPILLARY BUD FROM LIMBAL VESSELS (trend to neovascularization)		**2**
	Presence of inflammatory infiltrates, rolling in limbal vessel / conjunctiva zone	0>slight >50 cells/mm^2^	**2**
		50>mild> 100 cells/mm^2^	**4**
		100> moderate > 200 cells/mm^2^	**6**
		severe> 200 cells/mm^2^	**8**

In vivo confocal microscopy scale was obtained for five zones: the superficial epithelium, basal epithelium, and anterior stroma of the cornea, limbus, and conjunctival blood vessels. Cell morphology and nuclear aspects were evaluated, and the number of infiltrating inflammatory cells (lymphocytes, polymorphonuclear cells, or dendritic-like cells) was assessed.

We instilled 50 µl eye drops of sterile phosphate-buffered saline (PBS), 0.02% BAK solution (BAK Sol), 0.02% BAK in emulsion (BAK Em), 0.002% CKC solution (CKC Sol), or 0.002% CKC in emulsion (CKC Em) in rabbit eyes 15 times at 5 min intervals according to Ichijima et al. [29]. All the eye drops were supplied by Novagali Pharma (Evry, France) and were sterile with physiologic pH and osmolality. We compared 0.02% BAK to 0.002% CKC since these two QAC concentrations confer equivalent positive charge to the emulsion surface (zeta potential around 20 mV), and similarly enhanced ocular delivery could be obtained with cyclosporine A (CsA)-emulsions containing 0.002% CKC or 0.02% BAK (data not shown).

### Clinical findings and Draize test

The first instillation was chosen as time zero (T0). During the instillations, the time when conjunctival redness appeared was recorded. At H4, D1, and D4, the eyes were examined using slit lamp microscopy for ocular irritation and scored according to a weighted scale for grading the severity of ocular lesions modified from the Draize Test [25,26,28]. We especially evaluated the degree of redness, swelling (chemosis), and tearing of the conjunctiva; the degree and area of cornea opacity; and the increased prominence of folds and congestion of the iris. The possible maximum total score was 110 (conjunctiva=20, cornea=80, iris=10).

### In vivo confocal microscopy observation and scale

The laser scanning IVCM Heidelberg Retina Tomograph (HRT) II/Rostock Cornea Module (RCM; Heidelberg Engineering GmbH, Heidelberg, Germany) was used to examine the entire ocular surface [25-28]. The x-y position and the depth of the optical section were controlled manually; the focus position (µm) was automatically calculated by the HRT II/RCM. For all eyes, at least 10 confocal microscopic images of each layer in the conjunctiva/limbus/cornea were recorded and analyzed. The final scores were the averages of the 10 eyes of five animals.

An IVCM scale was established to quantify the ocular surface damage as presented in Table 1 [28]. Scores were obtained for five zones: the superficial epithelium, basal epithelium, and anterior stroma of the cornea, limbus, and conjunctival blood vessels. Cell morphology and nuclear aspects were evaluated, and the number of infiltrating inflammatory cells (lymphocytes, polymorphonuclear cells, or dendritic-like cells) was assessed by using the Cell Count® program (Heidelberg Engineering GmbH) associated with the HRT II/RCM. The maximal score was 40.

**Figure 1:**
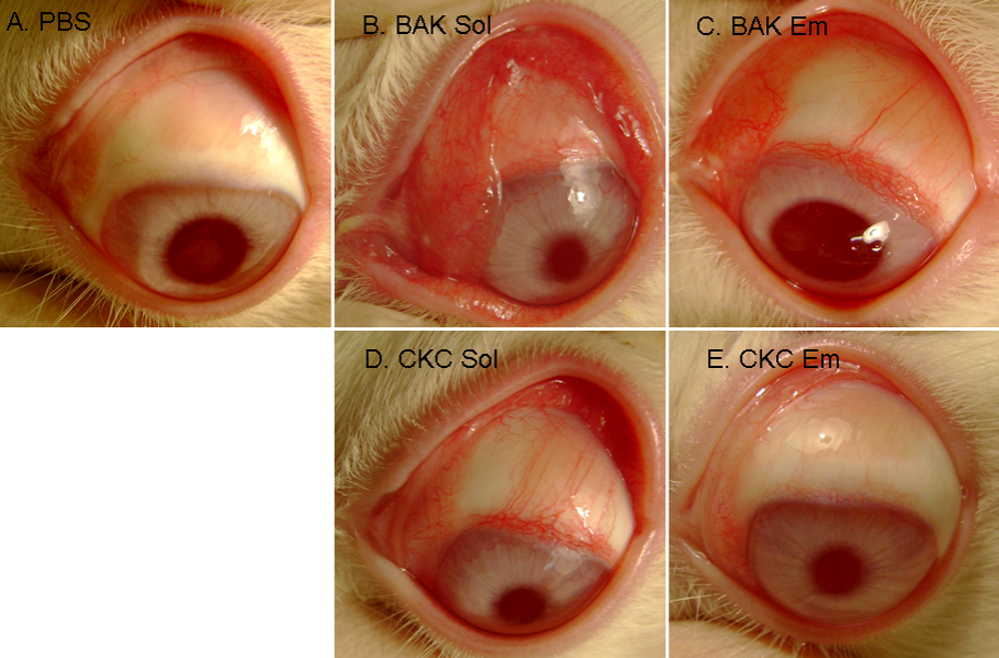
Microphotographs of typical clinical features. Microphotographs of typical clinical features of PBS- (**A**), BAK Sol- (**B**), BAK Em- (**C**), CKC Sol- (**D**), and CKC Em- (**E**) instilled rabbit eyes 4 h after repeated instillations are shown. BAK Sol induced diffuse hyperemia, chemosis, and purulent secretions on the conjunctiva. BAK Em and CKC Sol also induced mild conjunctival inflammation. CKC Em-receiving eyes presented no obvious abnormality on the conjunctiva and showed nearly the same aspect as the PBS-instilled eyes.

### Conjunctival impression cytology collection

IC specimens were collected by techniques previously described [25,27,28]. Two types of IC techniques were used for this study. Two nitrocellulose membranes (Millipore, Bedford, MA) were applied to the superior bulbar conjunctiva and then dipped into tubes containing 1.5 ml of cold PBS with 4% paraformaldehyde (PFA) for future cresyl violet cytology, and two Supor®-membrane (Gelman Sciences, Ann Arbor, MI) were dipped immediately after application into tubes containing 1.5 ml of cold PBS with 0.05% PFA and kept at 4 °C until FCM procedures.

### Cresyl violet staining of conjunctival impression cytology and morphological evaluation

The membranes dipped in 4% PFA were washed in distilled water, dehydrated into ethanol, and stained by cresyl violet solution (1%, number 5235, Merck, Fontenay-sous-Bois, France) for 30 min. The samples were then air-dried and mounted in a Eukitt medium (CML, Nemours, France).

We evaluated the morphology of the conjunctival ocular surface according to a modified Nelson’s classification [30], assessing the appearance of the epithelial cells (morphological changes in the cytoplasm and in the nucleus, the nucleocytoplasmic (N/C) ratio, and the metachromatic changes in the cytoplasm), inflammatory infiltration, and the density of goblet cells and subsequently assigning the grades to the ocular surface.

**Figure 2:**
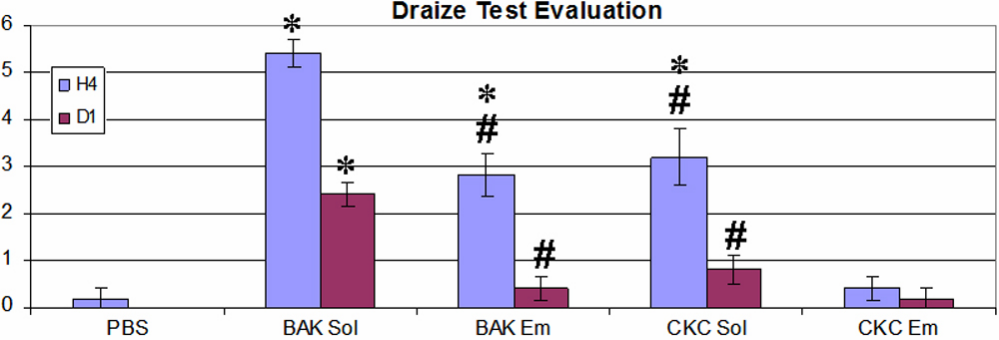
Draize test evaluation after PBS, BAK Sol, BAK Em, CKC Sol, and CKC Em instillations in rabbit eyes at H4 and D1. The asterisk indicates that p<0.01 compared to the PBS-instilled and CKC Em-instilled groups; the sharp (hash mark) denotes that p<0.05 compared to the BAK Sol-instilled group.

### Flow cytometry analysis of rabbit impression cytology specimens

Conjunctival cells were extracted as previously described [25,28]. Cells were extracted by gentle agitation and were analyzed on a flow cytometer (FC500; Beckman Coulter, Miami, FL). A direct immunofluorescence procedure was used to study the expressions of the class II antigen RLA (rabbit leukocyte antigen) DR (1:40; DakoCytomation, Clostrup, Denmark) and TNF-receptor 1 (mTNFR1, 1:40 dilution, R&D Systems, Minneapolis, MN). Mouse FITC-conjugated IgG1 (BD Biosciences PharMingen, San Diego, CA) was used as a negative control. For each antibody, a minimum of 1,000 conjunctival cells were analyzed, and the results were expressed as percentages of positive cells. Soon after the FCM analysis, we stained the cell suspension with propidium iodide (PI 0.5 µg/ml; Sigma Chemical Company, St. Louis, MO). Immunoreactive cells were then spun down on a glass slide using a cytospin centrifuge (Shandon Cytospin 4; Thermo, Electron Corporation, Waltham, MA) and later observed and photographed under a confocal microscope (E800; PCM 2000; Nikon, Tokyo, Japan).

### Cryosections and immunohistology

Two rabbits in each group were euthanized with a lethal dose of pentobarbital at D1. Enucleated eyes were fixed in 4% PFA and embedded. The 10 µm cryosections were incubated with antibodies directed against rabbit CD45 (1:50; CBL1412; Cymbus Biotechnology, Chandlers Ford, UK) to detect inflammatory cell infiltration. Sections were stained with secondary antibody and later with PI. To detect apoptotic cells, a terminal deoxynucleotidyl transferase-mediated dUTP-nick end labeling (TUNEL) assay (Roche Diagnostics, Meylan, France) was used. Cryosections were first permeabilized and then incubated with an apoptosis detection kit including the 10-μl TUNEL enzyme and 90-μlL TUNEL label at 37 °C for 1 h. After three washes in PBS, the slides were stained with PI.

Images were digitized using an Olympus BX-UCB fluorescent microscope (Olympus, Melville, NY) equipped with a DP70 Olympus digital camera and image analysis software. Positive cells to the different markers were counted in a masked manner in four different rabbit eyes in at least five areas.

### Statistical analysis

Results were expressed as means±standard error (SE). Draize and IVCM scores were compared using nonparametric comparisons (Mann–Whitney). The groups for analysis in IC expression with FCM and immunopositive cells counts were compared using factorial analysis of variance (ANOVA) followed by the Fisher’s method (Statview V; SAS Institute Inc., Cary, NC).

**Figure 3:**
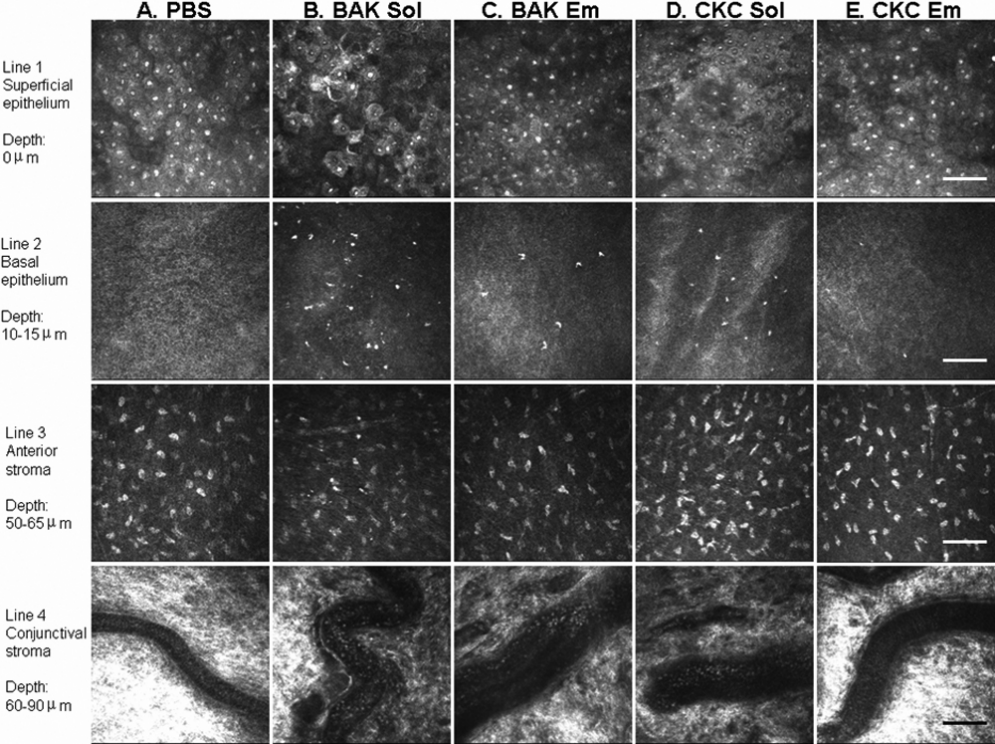
HRT II IVCM images of rabbit ocular surface. HRT II IVCM images of rabbit ocular surface after PBS (**A**), BAK Sol (**B**), BAK Em (**C**), CKC Sol (**D**), and CKC Em (**E**) instillations at D1 are displayed. Results are shown in the superficial epithelium (line 1), basal epithelium (line 2: 10–15 μm from the superficial epithelium layer), anterior stroma (line 3: 50–65 μm from the superficial epithelium layer), and conjunctival substantia propria (line 4: 60–90 μm from the superficial epithelium layer). BAK Sol-receiving eyes showed the greatest damage in the epithelium and the greatest inflammatory infiltration in the basal epithelium and anterior corneal stroma. BAK Em and CKC Sol induced intermediate toxicity. These three groups induced inflammatory cells rolling in conjunctival blood vessels. CKC Em presented almost the same aspects in all ocular surface structures as the PBS-instilled group. The scale bar indicates 100 μm.

## Results

### Clinical findings

Four hours after the first instillation (i.e., 2.75 h after the previous one), BAK Sol induced diffuse hyperemia, chemosis, and purulent secretions on the conjunctiva when compared with the PBS-instilled eye. BAK Em and CKC Sol also induced mild conjunctival hyperemia but less than what was induced by BAK Sol with no obvious chemosis or purulent secretion (Figure 1). CKC Em-receiving eyes (Figure 1E) presented no redness, chemosis, or secretions on the conjunctiva and showed nearly the same aspect as the PBS-instilled eyes.

PBS did not induce any redness during the instillation period. BAK Sol induced conjunctival redness very quickly 13±1.07 min after the first instillation (p<0.0001 compared to BAK Em and CKC Em). BAK Em and CKC Sol groups started to show visible redness at 34±2.08 min and 23±3.50 min, respectively, with no significant difference between the two groups. CKC Em induced a slight redness close to the end of the experiment 60±4.47 min after the first instillation (p<0.0001 compared to all other groups, except PBS).

### Draize test

At H4, the BAK Sol-, BAK Em-, and CKC Sol-instilled groups presented higher Draize Test scores than the PBS-instilled group (p<0.01 for the three groups; Figure 2). CKC Em presented no difference with the PBS group (p>0.05). The ocular toxicity score was the highest in the BAK Sol group (5±0.4), which had higher scores than the BAK Em (2±0.4), CKC Sol (3±0.6), and CKC Em groups (0.4±0.3; p<0.05 for the three groups). The BAK Em- and CKC Sol-instilled groups also showed higher ocular toxicity than the CKC Em-instilled group (p<0.01 for the two groups).

At D1, the PBS, BAK Em, CKC Sol, and CKC Em eyes all returned to normal aspects without significant differences among them. BAK Sol still induced substantial ocular abnormalities (p<0.01 compared to the PBS and CKC Em groups, p<0.05 compared to the BAK Em and CKC Sol groups). Only at D4 did the BAK Sol-instilled eyes return to a normal ocular aspect (data not shown).

**Figure 4:**
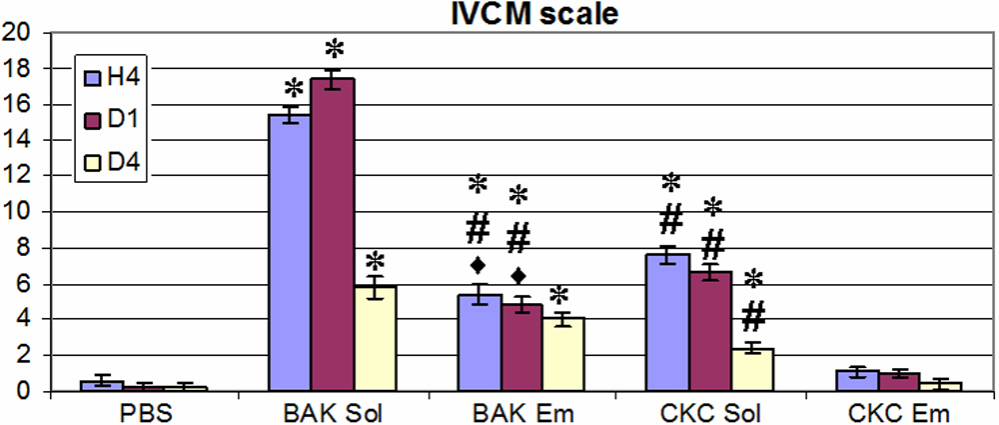
In vivo confocal microscopy scores in the five tested groups. The toxicity of CKC Em was less than that of BAK Sol, BAK Em, and CKC Sol with no significant differences with the PBS-instilled groups at all time points (H4, D1, and D4). BAK Sol presented the highest IVCM toxic score at H4 on D1 with intermediate results for BAK Em and CKC Sol. The asterisk indicates that p<0.01 compared to PBS and CKC Em; the sharp (hash mark) denotes that p<0.05 compared to BAK Sol; and the filled diamond symbol indicates that p<0.05 compared to CKC Sol.

### In vivo images of rabbit ocular surface after instillations

Figure 3 shows the IVCM images of the rabbit corneal epithelium (line 1), basal epithelium (line 2), anterior stroma (line 3), and conjunctival stroma (line 4) after application of PBS (Figure 3A), BAK Sol (Figure 3B), BAK Em (Figure 3C), CKC Sol (Figure 3D), and CKC Em (Figure 3E) at D1.

#### Surface epithelium

PBS-instilled rabbits presented almost a normal corneal epithelium (Figure 3A) with a regular polygonal mosaic appearance and brightly reflective nuclei. No obvious desquamation, swelling of epithelium, or inflammation was detected. BAK Sol (Figure 3B) induced partial desquamation of epithelial cells. The cells presented an irregular shape with abnormal reflectivity patterns and swelling cells, observed as a loss of cell borders. Inflammatory infiltrates were also found. Compared to the BAK Sol instillation, fewer abnormalities were observed for BAK Em (Figure 3C) and CKC Sol (Figure 3D) instillation with partial desquamation of epithelial cells and irregular cell shapes. The CKC Em group (Figure 3E) showed almost the same epithelial aspects as did PBS-instilled rabbits without obvious epithelium abnormality or inflammatory infiltration.

#### Basal epithelium

PBS (Figure 3A) and CKC Em (Figure 3E) induced no obvious inflammation in this layer whereas BAK Sol (Figure 3B) induced the greatest infiltration (129±13.29 inflammatory cells/mm^2^, p<0.001 compared to all other groups). These bright hyperreflective inflammatory infiltrates were also found at a moderate level in the BAK Em-instilled eyes (Figure 3C; 55±6.00 inflammatory cells/mm^2^, p<0.001 compared to CKC Em) and in the CKC Sol-instilled eyes (Figure 3D; 55±11.12 inflammatory cells/mm^2^, p<0.001 compared to CKC Em).

#### Anterior stroma

One day after instillation of BAK Sol (Figure 3B), slight inflammatory infiltration and slight disorganization of the anterior stroma was recorded by IVCM. No abnormality was observed in the anterior stroma in the other groups (Figure 3A,C–E).

**Figure 5:**
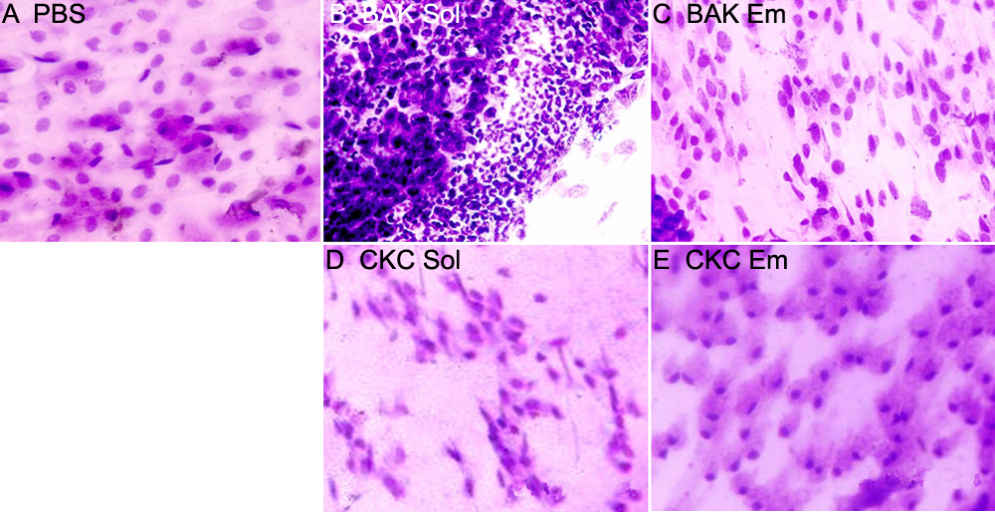
Conjunctival impression cytology stained by cresyl violet at D1. PBS (**A**) presented normal aspects of the conjunctival epithelium with no inflammatory infiltration. BAK Sol (**B**) induced numerous polymorphonuclear inflammatory cells with almost no normally shaped epithelial cell visible. BAK Em (**C**) and CKC Sol (**D**) both showed epithelial damage with inflammatory infiltration. CKC Em-instilled (**E**) rabbit eyes presented normal epithelial patterns without inflammatory infiltration. (original size 40×).

#### Posterior stroma and endothelium

At all the observation times, no abnormality was observed in any group (data not shown).

#### Limbus

Minimal inflammatory cells were observed after PBS and CKC Em instillations. In the BAK Sol-instilled group, we observed that the inflammatory infiltrations in the peripheral cornea and limbus area were more abundant than in all the other groups (data not shown). We also observed the presence of capillary buds from limbal vessels at this time. Moderate inflammatory infiltration was also observed in the BAK Em and CKC Sol groups.

#### Conjunctiva

Blood vessels in hyperreflective conjunctiva were observed by IVCM. The PBS-instilled rabbit presented normal conjunctival blood vessel aspects with no rolling inflammatory cells (Figure 3A). After the applications of BAK Sol (Figure 3B), BAK Em (Figure 3C), and CKC Sol (Figure 3D), inflammatory cells rolling along vascular walls were consistently recorded in blood vessels. In contrast, CKC Em-instilled eyes (Figure 3E) presented almost normal blood vessel aspects as did PBS-instilled rabbits with no obvious rolling cells. At H4 (images not shown), IVCM showed the same tendency of toxic ranking with BAK Sol inducing the worst aspect of epithelium and the greatest inflammatory infiltration; BAK Em and CKC Sol induced moderate abnormalities in cornea, limbus, and conjunctiva; and the CKC Em group showed almost the same images as did the PBS-instilled group. At D4 (images not shown), the abnormalities found in the ocular surface decreased in all groups. BAK Sol, BAK Em, and CKC Sol groups still presented abnormal aspects in limbus and conjunctival blood vessels. These slight abnormalities disappeared at D7 after instillations (data not shown). According to the IVCM observations, the CKC Em-instilled eyes presented no difference in the ocular surface compared to PBS-instilled eyes from H4 to the end of experiment.

### In vivo confocal microscopy scale evaluation

An IVCM scoring system was used to quantify toxic patterns. As shown in Figure 4, at H4 and D1, BAK Sol induced the highest IVCM score compared to PBS, CKC Em (p<0.01 compared to the two groups), BAK Em, and CKC Sol (p<0.05 compared to the two groups). At these time points, the BAK Em- and CKC Sol-instilled groups showed higher IVCM scores than did the PBS- and CKC Em-instilled groups (p<0.01 for the two groups). BAK Em eyes presented lower IVCM scores than did CKC Sol eyes (p<0.05) at H4 and D1. At H4, D1, and D4, the CKC Em-instilled group always presented similar IVCM scores to the PBS-instilled group with no statistical differences. At D4, scores decreased for every treatment except BAK Sol; BAK Em and CKC Sol still presented higher IVCM scores than the PBS (p<0.01 for the three groups) and CKC Em (p<0.01 for the three groups) groups. At D7, the IVCM scores of BAK Sol, BAK Em, and CKC Sol eyes returned to normal aspects (data not shown).

### Impression cytology staining

Impression cytology of conjunctival surface after PBS instillation at D1 showed normal, polyedric conjunctival epithelial cells (Figure 5A) with a prominent nucleus. The nuclear cytoplasmic ratio ranged from 1/2 to 1/3. There were no inflammatory infiltrating cells. The goblet cells were clearly visible among or beside the epithelial cells. In the BAK Sol-instilled group (Figure 5B), the conjunctival epithelium was barely recognized because of the very intense infiltration of polymorphonuclear cells. Goblet cells completely disappeared after BAK Sol treatment at this time. The lesions observed in conjunctival IC after cresyl violet staining are summarized in Table 2. BAK Em (Figure 5C) and CKC Sol (Figure 5D) induced moderate toxicity in conjunctival epithelium, which showed aspects of anisocytosis and anisonucleosis. Inflammatory infiltration was observed, and the density of goblet cells decreased after these two treatments. The CKC Em eyes (Figure 5E) presented a nearly normal conjunctival epithelium aspect with no obvious inflammatory infiltration. Goblet cells were clearly present with no morphological abnormalities.

**Table 2:** Description of cresyl violet conjunctival impression cytology results at D1 following repeated applications of quaternary ammonium compound emulsions and solutions

	PBS	BAK Sol	BAK Em	CKC Sol	CKC Em
Nucleocytoplasmic ratio	1/2 to 1/3	Not interpretable due to inflammation	1 to 1/2	1 to 1/2	1/2 to 1/3
Anisocytosis/ anisonucleosis	/	++++	++	++	+
Cell size	Normal	Very irregular	Irregular	Irregular	Normal
Inflammatory cells	/	++++	+++	++	/
Goblet cells	++	/	+	+	+++

Impression cytology of conjunctival surface after PBS instillation at D1 showed normal conjunctival epithelial cells without inflammatory infiltration. In the BAK Sol-instilled group, there existed intense infiltration of polymorphonuclear cells. The conjunctival epithelium and the goblet cells were barely recognized. BAK Em and CKC Sol induced moderate abnormality. The CKC Em eyes presented an almost normal conjunctival epithelium aspect without obvious inflammatory infiltration. Goblet cells were found to be normal. /: No abnormality, +: discrete, ++: moderate, +++: high, ++++: extremely intense

### Expression of rabbit leukocyte antigen (RLA) DR and tumor necrosis factor receptor type 1 (TNFR1) evaluated by flow cytometry

**Figure 6:**
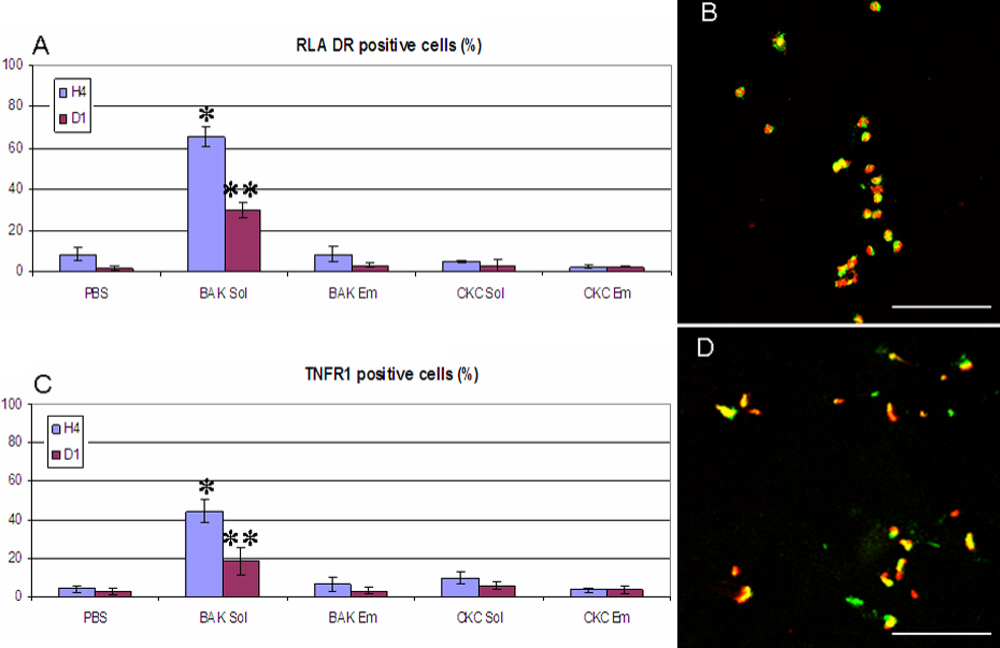
Impression cytology evaluated by flow cytometry and viewed after cytospin centrifugation. Percentages of RLA-DR- (**A**) and TNFR1- (**C**) positive cells after multiple instillations of PBS, BAK Sol, BAK Em, CKC Sol, CKC Em is displayed. The astersisk indicates that p<0.0001 compared with PBS, BAK Em, CKC Sol, and CKC Em-instilled groups, and the doube asterisk means that p<0.05 compared with PBS, BAK Em, CKC Sol, and CKC Em-instilled groups. Positive cells for RLA-DR (green, **B**) and TNFR1 (green, **D**) were viewed after propidium iodide staining (red) and cytospin centrifugation. The scale bar indicates 100 μm.

The baseline range of RLA DR- and TNFR1-positive cells in IC specimens from normal rabbits was approximately 3% - 6%. At H4, BAK Sol induced 65.4%±4.7% of cells positive for RLA DR (Figure 6A). These were mostly inflammatory cells (Figure 6B) as viewed after cytospin centrifugation. The RLA DR expression in the other groups was much lower than that of BAK Sol group with statistical significance (p<0.0001 compared to PBS, BAK Em, CKC Sol, and CKC Em groups). This high level of expression decreased one day after instillation; BAK Sol still induced 29.6%±3.7% of RLA DR-positive cells with significant differences with the other groups that showed normal levels (p<0.05 compared to the four other groups). At D4, RLA DR-positive cells returned to the normal level (approximately 5%) after BAK Sol instillation.

TNFR1 was also found at high levels at H4 after the instillation of BAK Sol (Figure 6C; 44.3%±5.9% of positive cells; p<0.0001 compared to all other groups). The cells positive to TNFR1 were mostly inflammatory cells as well as some typical conjunctival epithelial cells (Figure 6D). At D1, this strong expression of TNFR1-positive cells decreased to 18.2%±7.1%, which was still much higher than in the other groups (p<0.05 compared to all other groups). At D4, this expression returned to about 4% with no difference with all other groups.

### Immunostaining of CD45 and terminal deoxynucleotidyl transferase-mediated dUTP-nick end labeling markers in cryosections

**Figure 7:**
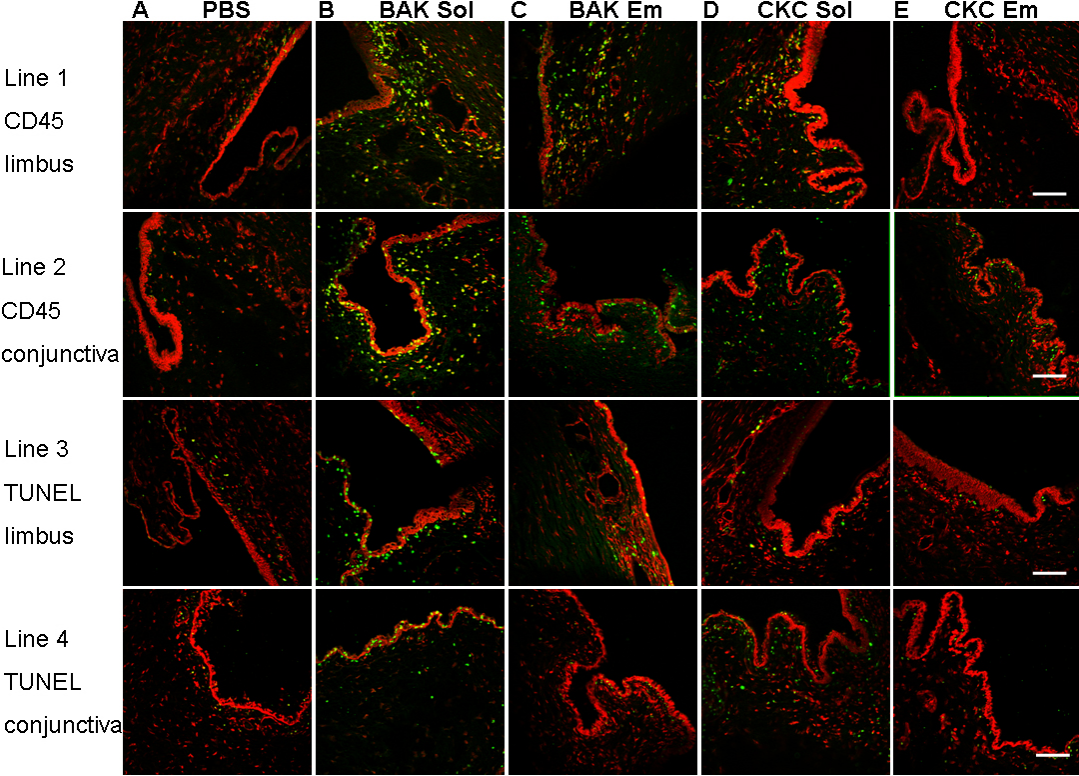
Immunostaining of inflammatory (CD45) and apoptotic (TUNEL) markers in cryosections. Immunostaining of inflammatory (CD45 in green: line 1 limbus, line 2 conjunctiva) and apoptotic (TUNEL in green: line 3 limbus, line 4 conjunctiva) markers in cryosections of rabbit eyes instilled with PBS (**A**), BAK Sol (**B**), BAK Em (**C**), CKC Sol (**D**), and CKC Em (**E**) at D1 is shown. Nuclei were stained in red with propidium iodide. The scale bars indicate 100 μm. Immunohistology clearly showed that BAK Sol instillation induced numerous CD45+ inflammatory cells and TUNEL+ apoptotic cells infiltrating the limbus and conjunctiva zones. BAK Em and CKC Sol also induced moderate inflammatory/apoptotic cells. After CKC Em or PBS instillation, occasional inflammatory/apoptotic cells were found.

**Figure 8:**
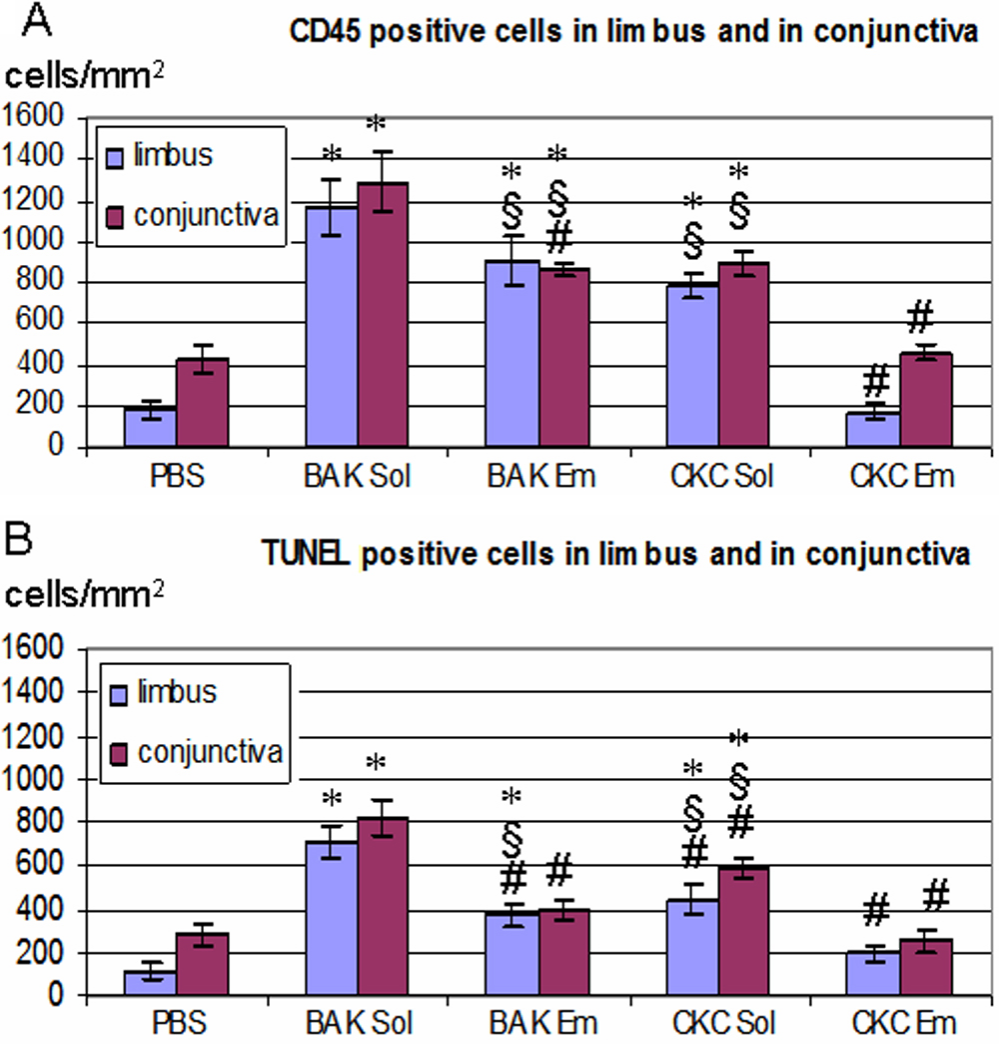
Counts of positive cells for CD45 and TUNEL markers. Counts of positive cells for CD45 (**A**) and TUNEL (**B**) after applications of PBS, BAK Sol, BAK Em, CKC Sol, or CKC Em at D1 are displayed in this graph. The asterisk means that p<0.005 compared to PBS; the sharp (hash mark) indicates that p<0.005 compared to BAK Sol; and the double “S” symbol (§) denotes that p<0.05 compared to CKC Em. A high level infiltration of CD45+ inflammatory cells and TUNEL+ apoptotic cells was found in the limbus and conjunctiva, especially in quaternary ammonium compounds solution-receiving eyes compared to quaternary ammonium compounds emulsion-instilled eyes.

Immunostaining of CD45+ inflammatory cells (line 1 for limbus, line 2 for conjunctiva) and TUNEL+ apoptotic cells (line 3 for limbus, line 4 for conjunctiva) at D1 are shown in Figure 7. The immunopositive cell counts are presented in Figure 8. PBS-instilled rabbits only showed a few CD45+ inflammatory cells in the limbus (Figure 7A; 180±43 cells/mm^2^) and conjunctiva (Figure 7A; 431±63 cells/mm^2^) zones. Immunohistology clearly showed that BAK Sol instillation induced numerous CD45+ inflammatory cells infiltrating the limbus (Figure 7B) and conjunctiva (Figure 7B) zones, 1160±134 cells/mm^2^ and 1290±139 cells/mm^2^, respectively (Figure 8A, p<0.005 compared to the PBS and CKC Em groups). These inflammatory cells were especially located in the limbal or conjunctival stroma but also beneath the epithelial layers. BAK Em presented moderate inflammatory infiltration, 900±121 cells/mm^2^ in the limbus (Figure 7C) and 860±34 cells/mm^2^ in the conjunctiva (Figure 7C; p<0.005 compared to PBS; and p<0.05 compared to CKC Em). BAK Em induced significantly fewer CD45+ cells than did BAK Sol (p<0.005). CKC Sol also presented moderate inflammatory infiltration (Figure 7D for limbus with 790±59 cells/mm^2^; Figure 7D for conjunctiva with 890±60 cells/mm^2^; p<0.005 compared to PBS and p<0.05 compared to CKC Em) with no difference with BAK Em treatment. After CKC Em instillation, occasional inflammatory cells were found in the limbal zone (Figure 7E, 170±40 cells/mm^2^) and beneath the conjunctival epithelium (Figure 7E, 460±34 cells/mm^2^) with no difference with the PBS group. In corneal tissue, very slight CD45+ expression was found in the BAK Sol-instilled group, and no other treatments induced inflammatory cells in the cornea (data not shown). Few apoptotic cells were observed in the limbal zone (Figure 7A, 110±38 cells/mm^2^) and in the conjunctiva (Figure 7A, 280±53 cells/mm^2^) after instillation of PBS. When BAK Sol was instilled in the ocular surface of rabbits, numerous apoptotic cells were found in the limbal zone (Figure 7B and Figure 8B, 710±82 cells/mm^2^) and conjunctiva (Figure 7B, 820±80 cells/mm^2^; p<0.005 compared to the PBS and CKC Em groups). Compared to BAK Sol, the BAK Em treatment induced fewer apoptotic cells in the limbal zone (Figure 7C, 370±47 cells/mm^2^, p<0.005 compared to the PBS and BAK Sol groups, p<0.05 compared to the CKC Em group) and in the conjunctiva (Figure 7C, 390±43 cells/mm^2^; p<0.005 compared to BAK Sol with no differences with PBS or CKC Em-instilled groups). Apoptosis was found in the limbal zone after CKC Sol application (Figure 7D, 440±69 cells/mm^2^); the conjunctival stroma also contained numerous apoptotic cells in subepithelial and deeper zones (Figure 7D, 590±41 cells/mm^2^; p<0.005 compared to the PBS and BAK Sol groups; p<0.05 compared to the CKC Em group). CKC Em induced fewer apoptotic cells in the limbal (Figure 7E, 190±35 cells/mm^2^) and conjunctival zones (Figure 7E, 250±48 cells/mm^2^) with no difference compared to PBS. In corneal tissue, few apoptotic cells were found in the superior epithelium of the cornea after BAK Sol and CKC Sol instillations. In other treatments, no apoptotic cells were found in the corneal layers (data not shown).

## Discussion

Lipid emulsions are known to improve the tolerance of topically applied ophthalmic drugs. Amphotericin B emulsion was found to be better tolerated than the commercial solution of Fungizone® in rabbits [31]. Several clinical studies of emulsion-based eye drops of artificial tears or cyclosporine showed good overall safety, efficacy, and comfort in normal subjects and dry eye patients [3,4,32,33]. Compared to other ophthalmic vectors, cationic vehicles have a better spreading capacity and improve ocular bioavailability [34-37]. Formulation of cyclosporine A in a cationic emulsion results in significantly improved (11 fold) corneal and conjunctival delivery in rabbits compared to olive oil [34]. Compared with the anionic emulsion, indomethacin in cationic emulsion provided significantly higher drug levels in the aqueous humor and sclera or retina [35], and submicron cationic lipid emulsion enhanced the ocular bioavailability of cyclosporine A [36]. The main mechanism involved is that cationic emulsions interact with the negatively charged ocular surface such as the cell membranes of the conjunctival and corneal epithelia. In an effort to optimize cationic emulsions, we proposed the use of QAC to provide the cationic charge. BAK cationic emulsion of cyclosporine significantly improved the penetration of cyclosporine over a negatively charged emulsion [4]. The use of lipophilic QAC, such as CKC rather than BAK, allows obtaining a positive charge with a lower amount of QAC because of its optimal oil/aqueous interface distribution. In the present study, we studied the toxicity of BAK and CKC cationic emulsions compared with their respective BAK and CKC solutions, and we observed a reduction of QAC toxicity when it was incorporated into the emulsion.

The model consisting of repeated applications in rabbit eyes (15 times at 5 min intervals) does not reflect the real ocular surface reactions in patients, but it may emphasize the action of low toxic compounds and mimic repeated administrations. As the repetition of instillations in a short period of time causes drastic stimulation of the ocular surface, this model is useful for between-drug comparisons and testing a specific compound’s absence of toxic effects, which is a good indicator of further absence of ocular toxicity in a more conventional use over the long-term. This model combined rapidity and efficiency in comparing the toxicity of several products without modifying their concentration and/or composition. It was therefore used in the past to distinguish the toxicity ranking after applications of 0.02%, 0.01%, and 0.005% BAK and clearly showed the different levels of epithelial deterioration [29]. In our study, this model also clearly demonstrated the differences between emulsion and solution formulations. Pertinent and reliable animal models are in great need for testing new formulations and new preservatives. To simulate long-term toxicity of QAC at reasonable time intervals and over a short period of time, previous studies have used high concentrations (50 fold to 500 fold the commercial concentrations) or repeated instillations over a long period of time to detect toxic effects [27,28,38], which may be complex in animal models. A short duration of testing, such as 14 or 28 days as used in standard toxicological evaluations, at commercial concentrations in young healthy animals may not reflect the clinical use when the drug is administered over the long-term sometimes in association with other drugs or preservative-containing eye drops or in patients with preexisting or concomitant ocular surface impairment. This point raises the problem of validating reliable tests for toxicological purposes and may explain why clinical trials often fail to show mild or subclinical toxic effects that are observed in patients treated for long periods of time, such as in glaucomatous patients in whom the ocular surface has widely shown inflammatory changes and clinical impairment [14-16,39-42].

Compared to the previous study by Ichijima [29], mainly describing the effects on the superficial corneal epithelium, we developed new in vivo tools and observed the entire ocular surface, the cornea, the limbus, and the conjunctiva, at various depths. Moreover, we studied inflammatory markers in conjunctival imprints and confirmed this analysis by using standard immunohistology after animal sacrifice. This approach provides much broader and more reliable information than clinical assessment alone as in the standard Draize test or even in investigations at the corneal level alone as conjunctival inflammation may not be assessed by corneal-based examinations. Moreover, this complementary set of investigations decreases the need for large animal series as IVCM or conjunctival IC may be used repeatedly for monitoring drug toxicity or efficacy.

The toxicity ranking of the four eye drops tested in our study was distributed as follows BAK Sol > BAK Em ≈CKC Sol > CKC Em, this latter formulation being almost nontoxic in our experimental conditions. Interestingly, when formulated in an emulsion, both BAK and CKC presented reduced toxicity compared to the same concentration in solutions. This reduction of toxicity of BAK/CKC in emulsion is attributed to the distribution of the QAC within the oil phase, leading to a low concentration of QAC in the aqueous phase. One of the proposed mechanisms of QAC for antimicrobial activity is their intercalation within the bacterial membrane [43]; in emulsion, they behave in a similar way by distributing at the emulsion droplet surface. We therefore suggest that the QAC that is bound to the emulsion surface is not available for binding with bacterial membranes and that only the remaining free QAC is responsible for the preservative effect and the toxicity. In the formulation tested in the present study, eventual toxicity was reduced not only by the use of cationic emulsion but also by selecting a highly lipophilic QAC such as CKC to improve the compound partition within the emulsion. The lipophilicity of CKC partition is almost 100-fold more important in octanol than in water, which is four to five times higher than BAK. Compared to BAK, CKC distributed even more preferentially into the oil phase (allowing reduced cationic agent content) with a lesser amount dissolved in the water phase and free for possible toxic damage. Moreover, QAC could enhance membrane fluidity, and CKC was found more efficient than BAK [44]. In our experiments, CKC Em exhibited almost the same aspect as the PBS-instilled group and did not induce any obvious ocular toxicity during all the observation times. Consequently, CKC-associated emulsions present the advantages of cationic emulsions and reduced free QAC and demonstrated no obvious toxicity. Pharmaceutical companies require new in vivo and in vitro tools to test the possible toxicity of their newly manufactured drugs. In vivo tests present the advantages of mimicking the real ocular environment, especially regarding the composition of the lachrymal film and drug metabolism in ocular tissues. At the same time, animal experimentation guidelines require refining the tests to reduce the number of animals used. In addition to demonstrating the tolerance of emulsion-containing eye drops, this study has also proved that it is possible to refine the classical scoring elaborated by Draize test, based on clinical evaluations in 1944. However, as the gold standard in ocular toxicology, it lacks precise and objective criteria, especially at cellular levels. The Draize test is therefore more of a good standard for eliminating truly toxic drugs in a screening approach than in a predictive evaluation of the real use of eye drops in further clinical development. We developed both the IVCM and flow cytometry on IC for our toxicological models. Used in combination, each of these techniques was able to detect and analyze the microstructures of the animal’s ocular surface (cornea, limbus, and conjunctiva) as well as the surface markers expressed by conjunctival epithelium in toxic conditions. Based upon histological precise patterns of the ocular surface for three-dimensional visualization, IVCM offers the advantage of examining the same animal in vivo during experimental procedures and could not be replaced with any other standardized method except histology after sacrifice, which would have required a much higher number of animals to provide successive time points. Moreover, specifically in the rabbit model, IVCM also allows us to explore the limbus and conjunctiva blood vessels that are difficult to see in small eyes such as rat eyes. The IVCM scale could also provide a classification of global toxicity in the cornea, limbus, and conjunctiva. This scale system could be used as an effective tool for evaluating ocular surface toxicity in a standardized way in many laboratories worldwide.

Conjunctival IC also usefully completed the information provided by IVCM. IC specimens were evaluated using a standard cytology method to identify the different cell populations in the conjunctival epithelium and then using FCM to assess the levels of inflammation or apoptosis. Thus, IC specimens were also efficient ex vivo tools for evaluating and comparing different toxic agents. As for human eyes [45], rabbit conjunctival imprints collect enough cells to ensure reliable results obtained from FCM. In our study, RLA-DR and TNFR1 were two inflammatory markers that were found to be significantly increased after toxic injuries induced by BAK Sol, confirming local inflammatory infiltration even one day after instillation.

In this study, we proposed a set of tools for exploring a drug’s toxicity in all components of the ocular surface, and this set is more pertinent, complete, and reliable than if those tools were used individually as in former studies. We also showed that the use of long-chain CKC-cationic emulsions should be further developed in eye drops because of their reduced toxicity, the improvement in drug ocular delivery, and finally for the comfort brought to patients.
